# Zebrafish as a Model to Evaluate Nanoparticle Toxicity

**DOI:** 10.3390/nano8070561

**Published:** 2018-07-23

**Authors:** Enamul Haque, Alister C. Ward

**Affiliations:** 1 School of Medicine, Deakin University, Waurn Ponds, VIC 3216, Australia; md.haque@deakin.edu.au; 2 Centre for Molecular and Medical Research, Deakin University, Waurn Ponds, VIC 3216, Australia

**Keywords:** zebrafish, nanoparticles, nanotoxicity, biocompatibility

## Abstract

Nanoparticles are increasingly being developed for in vivo use, from targeted drug delivery to diagnostics, where they have enormous potential, while they are also being used for a variety of applications that can result in environmental exposure for humans. Understanding how specific nanoparticles interact with cells and cell systems is essential to gauge their safety with respect to either clinical or environmental exposure. Zebrafish is being increasingly employed as a model to evaluate nanoparticle biocompatibility. This review describes this model and how it can be used to assess nanoparticle toxicity at multiple levels, including mortality, teratogenicity, immunotoxicity, genotoxicity, as well as alterations in reproduction, behavior and a range of other physiological readouts. This review also provides an overview of studies using this model to assess the toxicity of metal, metal oxide and carbon-based nanoparticles. It is anticipated that this information will inform research aimed at developing biocompatible nanoparticles for a range of uses.

## 1. Introduction

### 1.1. Nanoparticles

According to the definitions of the International Organization for Standardization, American Society of Testing and Materials and National Institute of Occupational Safety and Health, particles with a diameter between 1 and 100 nm or fibers spanning the range 1–100 nm are termed as nanoparticles (NPs) [1]. Over the last decade, the synthesis, analysis and application of nanoparticles has grown exponentially, becoming an active area of intense innovation [2]. Recent advances allow for the synthesis of nanosized particles with multiple properties, referred to as multifunctional nanoparticles, which includes metal and metal oxides nanoparticles, fullerenes, carbon nanotubes (CNT), nano polymers and crystalline materials [3,4].

### 1.2. Applications of Nanoparticles

NPs are being utilized for a wide range of applications. In manufacturing they are employed as chemically-inert additives, from fillers to pigments to anti-caking agents, but increasingly to create functional surfaces/membranes with catalytic, anti-microbial, UV protection, filtration and other diverse properties [5]. They are also fueling a new discipline of nanomedicine, lying at the intersection of chemistry, physics and medicine and focused on a range of biomedical applications. These include use as biosensors for nucleic acids, metabolites, proteins, drugs, pathogens and cancer cells by taking advantage of inherent optical, electrochemical, piezo-electric and photoluminescence properties [5,6]. NPs are also being used in a broad range of bioimaging, drug delivery, tissue engineering and other therapeutic applications, such as photoablation, which are facilitated by the accessibility of these small molecules throughout the body, and the ability to link with specific targeting modalities as well as carry a functional payload [4,6,7].

### 1.3. Measuring Nanoparticle Toxicity

Biomedical applications explicitly require nanoparticles that are non-toxic. However, toxicity is also an important consideration for nanoparticles used for manufacturing and other applications, since these can result in environmental exposure [2,8]. A number of different platforms are available to assess toxicity, ranging from in vitro cell culture assays to basic model organisms, such as sea urchin and daphnia, to advanced higher vertebrate models, like rodents and primates [9]. Cell lines and simple organisms are useful for cell-level toxicity and genotoxicity studies, but higher vertebrates are essential to detect complex physiological interactions. However, rodent models are high cost, have relatively slow and inaccessible embryo development, require substantial amounts of material for testing due to their relatively large size, and are accompanied by ethical concerns about their use—while primate models have similar issues, but to an even greater extent [9]. Therefore, small, low cost but sophisticated models are very attractive for the evaluation of in vivo nanotoxicity. In this context the zebrafish serves as a compelling, efficient and cost-effective alternative [10].

## 2. Zebrafish as a Model

### 2.1. Overview

The zebrafish (*Danio rerio*) is an established vertebrate model for the study of development and disease [11,12,13,14,15,16] and is being increasingly used for both pre-clinical studies and toxicological applications due to a range of favorable traits [17,18]. Zebrafish require relatively inexpensive housing, making them very cost-effective, and are small in size, reducing housing requirements as well as the quantity of agent required for testing [11,16]. They also exhibit a high fecundity rate, with a single female able to produce around 300 eggs, further underpinning their efficiency as a model [11,19]. The zebrafish and human genomes share ~70% similarity [20,21]. There is also very good conservation of major developmental and physiological processes, with key organ systems, such the digestive, nervous and cardiovascular systems, similar to humans [22]. This largely underpins the extensive equivalence in response to pharmacological agents between the two species [23], with many zebrafish models mimicking human diseases both genetically and phenotypically [16].

### 2.2. Zebrafish Development

**Figure 1:**
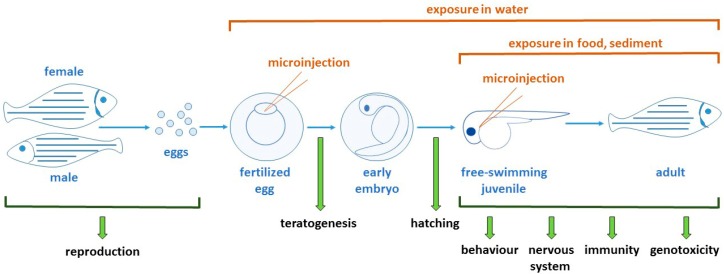
Toxicological studies performed in zebrafish. Schematic representation of zebrafish development, from spawning of embryos through their rapid development and hatching into free-swimming larvae and further growth and development into adults. Nanoparticles (NPs) can be administered via a variety of routes, including injection into eggs or specific sites on juveniles and adults, or alternatively administered in the water, sediment or food. The key assays used to examine toxicity in this model are indicated.

Zebrafish eggs are robust and develop externally, making them easy to manipulate and amenable to high-throughput applications. This is further augmented by the optical transparency of the developing zebrafish, which allows exquisite visual analysis, including of fluorescent and other markers [12,16]. Development is also incredibly rapid, with the basic zebrafish body plan well-established by 24 h post fertilization (hpf), with embryogenesis completed by 72 hpf and most organs fully developed by 96 hpf and reaching adulthood in around 3 months [12]. This makes them amenable to a wide variety of toxicological applications throughout their lifespan (Figure 1).

## 3. Approaches for Measuring Nanoparticle Toxicity in Zebrafish

### 3.1. Assessing Teratogenic and Other Developmental Effects

The rapidly developing, transparent and external embryos of zebrafish [24] are ideally suited for screening for agents that disrupt normal development, from the rapid cell divisions that follow fertilization, to the extensive morphogenesis that occurs during epiboly to the development of the body plan and its key organs and other structures [25,26]. Particularly sensitive to perturbation are the eye, brain, heart, notochord and fin, while visualization of the effects on pigmented cells, including red blood cells, as well as hatching and overall mortality, is very easy. For example, disrupted eye development and pigmentation was observed via simple light microscopy following exposure of zebrafish embryos to functionalized gold nanoparticles [27]. In addition, the dose- and time-dependent toxicity of silica NPs was able to be readily gauged by assessing mortality rates [28] and impacts on the cardiovascular system [29]. Furthermore, the enhanced biocompatibility of chitosan NPs compared to normal chitosan particles was evaluated by quantifying relative hatching [30].

### 3.2. Immunotoxicity

The immune system has been shown to be very sensitive to a variety of agents, including NPs, particularly the induction of an inflammatory response as well as associated accumulation and activation of neutrophils and macrophages [31]. For example, gold (Au) NPs have been shown to disrupt pathways involved in inflammatory and other immune responses [32], while silver (Ag) NPs caused immunotoxicity in adult zebrafish due to oxidative stress [33].

### 3.3. Genotoxicity

DNA is susceptible to damage following exposure to many chemical entities, which results in gene mutations and larger chromosomal alterations, collectively termed genotoxicity [34]. This can be assessed in embryos, larvae or adult tissues in a number of approaches, including quantitative RAPD-PCR methodology that has been used to demonstrate dose-dependent genotoxicity of TiO_2_ NPs [35], and comet assays for examining the impact of ferric oxide (Fe_2_O_3_) NPs [36].

### 3.4. Reproduction Analysis

The high fecundity rates of zebrafish are well suited to assessing the impact of agents on various aspects of reproduction, from egg production to fertilization rates to subsequent embryo viability. For example, reduced egg production and increased embryo mortality represent a hallmark of chronic exposure with TiO_2_ NPs [37], while in contrast Ag NPs mediated enhanced maturation of zebrafish oocytes due to increased oxidative stress and resultant follicle cell apoptosis [38].

### 3.5. Neurotoxicity and Behavioral Analysis

Zebrafish exhibit a range of complex behaviors that can be used as sensitive parameters to assess toxicity, including color preference, spatial recognition and locomotion [39,40]. The nervous system that underpins these behaviors, including the developing brain, is particularly vulnerable to oxidative stress because of its high energy demand, low level of antioxidants, and high cellular content of lipids and proteins. A range of NPs can trigger free radical activity at their surface, thereby creating oxidative stress at the site of particle deposition and translocation [41,42,43]. Neurotoxicity has been commonly noted for NPs that are able to reach the brain, where they can lead to neurodegeneration [44,45]. Behavioral effects have also been seen that are specific for particular NPs. For example, altered color preferences were found to be caused by silicon dioxide (SiO_2_) NPs [46], while locomotor activity was affected by cadmium telluride (CdTe) quantum dots [47]. More detailed analysis has identified enhanced neuron apoptosis and glial cell proliferation, along with altered gene expression, following exposure to titanium dioxide (TiO_2_) NPs [48].

### 3.6. Other Approaches

A range of additional techniques can be used to further understand potential toxicity, including a variety of high-content approaches [49], such as transcriptome analysis [36,50] and plasmonic spectroscopy [51]. Taking advantage of the optical transparency and genetic amenability of zebrafish, transgenic reporter lines have increased the ease and efficiency of toxicology studies [47,52]. Certain agents have inherent luminescent or fluorescent properties that can also be exploited to provide dynamic imaging of zebrafish embryos [53], including studies examining NP distribution in vivo [54,55].

### 3.7. Comparison between Zebrafish and Mammalian Studies

A number of studies have demonstrated that a variety of compounds tested in zebrafish embryos yielded similar results to those observed in rodents. This includes research showing comparable developmental toxicity for a series of 1,2,4-triazoles [56] and organotins [57], as well as correlations between zebrafish embryo LC50 (lethal concentration 50%) values and rodent LD50 (lethal dose 50%) values for a set of 60 compounds [49], although the latter study showed that the compound class influenced relative toxicity [49]. In a landmark study, a meta-analysis of toxicity testing in zebrafish of over 600 chemicals was performed, which compared aggregated toxicological end-points with outcomes observed in laboratory mammals. This demonstrated that for acute toxicity, there was a good correlation between zebrafish embryo LC50 values and LD50 values obtained across various laboratory mammals when the chemicals were administered via inhalation or injection, but not orally [58], which was consistent with other studies [59,60]. There was also a correlation between effects on zebrafish hatching and pre-natal loss in rabbits [58]. Furthermore, analysis of teratogens using zebrafish correctly ranked known mammalian teratogens [61,62]. Of note, zebrafish embryos were consistently more sensitive than mammalian systems to the agents tested [58,59].

### 3.8. Standardization of Zebrafish Testing

In light of the effectiveness of using zebrafish and other fish species for toxicity studies, test guidelines have been developed by the Organization for Economic Cooperation and Development (OECD). These include guidelines for the Fish Embryo Toxicity (FET) test [63], Fish: Juvenile Growth Test [64], and the adult-based Fish: Acute Toxicity Test [65]. The FET test has been validated by independent laboratory testing and has been shown to be a robust and reproducible methodology [66]. It has also been shown to have an excellent correlation with the Acute Toxicity Test in adults [67], and has been proposed to be a worthy range-finder prior to more extensive testing [68]. A recent study used the FET test to evaluate toxicity of methylxanthine drugs, which revealed a strong positive correlation between TC50 measurements of mortality, morphological defects and teratogenicity in zebrafish embryos and published mammalian LD50 values [69].

## 4. Selected Nanotoxicology Studies in Zebrafish

Zebrafish have been utilized for a myriad of nanotoxicology studies [36,37,38,48,70,71]. A summary of key studies involving the analysis of metal, metal oxide and carbon-based nanoparticles, particularly those related to shape, size and surface charge are discussed below, with relative toxicity summarized (Table 1).

### 4.1. Metal Nanoparticles

#### 4.1.1. Gold

Among metal nanoparticles, Au NPs have been the most widely studied in biomedicine, with key applications as drug carriers [83] and as diagnostic agents [84]. However, Au NPs can cause cytotoxicity in humans [85,86]. As a consequence, zebrafish has become increasingly used as an in vivo model to evaluate the toxicity of Au NPs.

Food containing Au NPs (12 and 50 nm) elicited a variety of cellular dysfunctions as well as genome alterations in adult zebrafish that were dependent on size, concentration and exposure time [87]. Chronic exposure of zebrafish to sediment containing 14 nm Au NPs also resulted in genome modification in various adult tissues, probably related to increased oxidative stress [88]. Au NPs were found to accumulate within tissues, suggesting the potential for higher toxicity compared with ionic Au. This was supported by other work that demonstrated 10–50 nm Au NPs could induce strand breaks in zebrafish ovaries, in addition to other cell types [89].

Surface charge functionalization has been identified as a critical determinant of toxicity. In one key study, Au NPs functionalized with positively-charged N,N,N-triethylammoniumethanol (TMAT) caused significant mortality, but elicited negligible malformations, whereas those functionalized with negatively-charged 2-mercaptoethanatesulfonate (MES) substantially increased the incidence of malformations, but did not result in significant mortality within the five-day exposure period. Neutral 2,2-mercaptoethoxyethoxyethanol (MEEE) and 2,2-mercaptoethoxyethanol (MEE) functionalized Au NPs did not elicit adverse effects even at higher concentrations of up to 250 ppm. A related study additionally found that exposure to either positively and negatively charged Au NPs resulted in behavioral abnormalities, including hypo-locomotor activity [90]. Kim et al. further showed that 1.3 nm TMAT-functionalized Au NPs caused abnormal eye development, with altered pigmentation and neuronal damage and concomitant behavioral changes [27]. These effects correlated with the ability of charged Au NPs to mediate inflammatory and altered immune effects [32].

Shape represents another important factor mediating the effects of Au NPs. The toxicity and biodistribution of fluorescently-labeled Au NPs of different shapes were examined in adult zebrafish [91]. Rod-shaped Au NPs exhibited enhanced uptake and clearance, while star-shaped Au NPs in contrast displayed slower uptake but longer sequestration in comparison to rod-shaped or spherical particles.

This study also showed that the choice of linker—polyethylene glycol (PEG) or mannose-capped—also had a significant effect [91]. Other research with peptide-capped Au NPs showed that the terminal modification was important, with the presence of terminal histidines being more toxic than tryptophans, with methionine conferring the least toxicity [92]. In addition, co-incubation of zebrafish embryos with Au NPs and a surfactant (polysorbate 20) resulted in increased uptake and toxicity [93]. Together this suggests that the overall shape and surface chemistry of the Au NPs are key determinants of biocompatibility.

#### 4.1.2. Silver

Ag NPs have also been extensively studied, with broad applications as therapeutic agents [94], antimicrobial agents [95], drug delivery systems [96] and biosensors [96].

Exposure of zebrafish to Ag NPs during early development elicited a range of toxicities, including a reduction in heart rate, damage to neuromast hair cells and more modest but statistically significant increases in both mortality and teratogenicity [97]. Another study demonstrated that low concentrations of 10–20 nm Ag NPs (<5 mg/L) had little impact on normal embryonic development, but higher concentrations resulted in significant effects on the development of mesodermal and ectodermal tissues, possibly due to delayed or inhibited cell division [98]. Exposure of adult zebrafish to Ag NPs resulted in localization in the gills and liver, where they caused oxidative stress and immunotoxicity [33].

The size-dependence of Ag NP-mediated toxicity is still debatable. One study reported similar mortality for zebrafish embryos treated with Ag NPs across a size range of 3–200 nm [99]. In contrast, Lee et al. showed that Ag NPs with a particle size of 30–72 nm were able to selectively enter zebrafish embryos through chorionic pores via random Brownian motion and caused more potent toxic effects [70]. In another study, Ag NPs were found to impact on neural development of zebrafish embryos in a size-dependent manner. In this case, 4 nm Ag NPs were taken up more efficiently than 10 nm-sized particles, with the head of the exposed zebrafish embryos able to accumulate more Ag NPs than the trunk [100].

The charge characteristics of Ag NPs has also been demonstrated to be an important determinant of toxicity. Investigation of ~12 nm Ag NPs functionalized with peptides of different charge revealed that positively-charged Ag NPs were the most biocompatible, with the extent of deformity and level of mortality greater in those embryos exposed to NPs with a negatively-charged peptide coating and greatest for those with a highly negatively-charged peptide coating [101].

Ag NPs of several different shapes were all shown to induce oxidative stress, but plate shaped Ag NPs were more toxic than spherical and wire-like shaped forms [102,103]. Interestingly, these effects correlated with the presence of surface defects rather than Ag shedding [102]. Coating with cysteine [102] or sulfidation [104] resulted in Ag NPs that elicited reduced oxidative stress in embryos or adults, respectively. Other studies have further demonstrated that embryonic toxicity of Ag NPs was augmented by exposure to simulated solar light [105]. Collectively, this suggests a complex interplay of factors, where a range of physiochemical properties underpin biocompatibility.

#### 4.1.3. Cadmium-Based Quantum Dots

Quantum dots (QDs) have been extensively employed for biological and medical imaging because of their small size and bright fluorescence, with broad absorption spectra, narrow emission spectra and high photostability [101,106,107,108]. Cadmium selenide (CdSe) and CdTe nanocrystals represent two of the most commonly utilized QDs for biological applications, with their extremely bright fluorescent properties making them powerful labelling agents for diverse in vivo applications. Several studies have shown CdSe QDs to be well tolerated with only modest toxicity even when directly injected into embryos at relatively high concentrations [47,109]. However, exposure of CdTe QDs to zebrafish embryos lead to a range of developmental and behavioral disturbances [75].

### 4.2. Metal Oxides

Metal oxide NPs, and particularly those based on TiO_2_, are widely used in a range of products, including paint, food and personal care products [110,111]. Due to concerns over the possible consequences of environmental exposure, their biocompatibility has been extensively studied using zebrafish.

#### 4.2.1. TiO_2_

Low dose (1 mg/L) exposure of zebrafish embryos to TiO_2_ NPs failed to induce major developmental malformations [112], although several groups have demonstrated that TiO_2_ NPs cause premature hatching in a dose-dependent manner [113,114]. In addition, higher doses of TiO_2_ NPs can lead to embryonic malformation and death [45]. Another study demonstrated that TiO_2_ NPs were able to absorb photons and produce electron-hole pairs that interact with water and oxygen to form reactive oxygen species that were toxic to zebrafish larvae [115]. Chronic exposure of adult zebrafish to TiO_2_ NPs at low concentrations (<4 mg/L) for 6 months was also associated with low toxicity, as determined by mortality rate. However, at higher concentration these NPs were found to accumulate in different parts of the fish, including the gill, liver, heart and brain [116]. High level exposure can also lead to genotoxic effects [35].

However, the major effect of TiO_2_ NPs is neurotoxicity. Even low levels of TiO_2_ NPs showed impacts on embryonic neurogenesis and neuronal differentiation observed [112], while exposure of larvae to TiO_2_ NPs significantly affected swimming parameters, including average and maximum velocity [117]. TiO_2_ NPs were able to cross the blood-brain barrier to damage the brain [45]. Chronic exposure of adult zebrafish to low dose TiO_2_ NPs for 45 days led to reduced levels of neurotransmitters and consequent changes in behavior, as well as histopathological changes in the zebrafish brain, which were associated with dose-dependent increases in nitric oxide levels [48]. Other researchers have compared the effect of bulk TiO_2_ and TiO_2_ NPs on the zebrafish brain, which revealed that TiO_2_ NPs were more toxic than bulk TiO_2_ due to enhanced lipid oxidation and degradation [118].

#### 4.2.2. Zinc Oxide (ZnO)

Zinc oxide (ZnO) NPs (20 nm) have also been shown to cause delayed development and inhibition of hatching in zebrafish embryos but only at higher concentrations (>0.1 mg/L) [77,119]. Again, surface properties and shape appear to be important. Polymer-coated ZnO NPs were shown to be more biocompatible than spherical ZnO, with leaf-shaped ZnO NPs having the greatest impact on hatching [120]. In another study comparing different shapes of ZnO NPs, nanosticks were found to be more toxic than nanospheres and cuboidal submicron particles with respect to hatching and overall mortality [121]. However, similar results were observed between ZnO NPs and bulk ZnO, suggesting that leaching of the metal oxide may be a key factor in mediating the effects of ZnO NPs [77]. Co-incubation with humic acid was able to suppress the impact of the released zinc [119].

#### 4.2.3. Other Metal Oxides

A number of studies have shown that other metal oxide NPs elicit variable toxicity. For example, magnesium oxide (MgO) NPs (20 nm) decreased hatching rate and survival of zebrafish embryos in a dose-dependent manner, leading to various types of malformations [78]. Fe_2_O_3_ NPs caused severe deformities in embryos [122] and elicited strong genotoxic effects in adult zebrafish [36]. Others have observed that NPs based on cupric oxide (CuO) and nickel oxide (NiO) also interfered with hatching, whereas those utilizing cobalt oxide (Co_3_O_4_) [123] or aluminum oxide (Al_2_O_3_) [77] were relatively inert.

### 4.3. Carbon Based Nanoparticles

Carbon-based nanomaterials have attracted increasing interest in the field of biomedical research, including as drug delivery systems, tissue scaffold reinforcements and cellular sensors [124]. Carbon NPs are considered particularly promising due to their low toxicity compared to other NPs [125]. In the last few years, the toxicity of different carbon NPs have been evaluated using zebrafish as a model, such as fullerenes, carbon nanoparticles, carbon nanotubes (CNT), graphene QDs and carbon QDs (C-dots).

#### 4.3.1. Fullerenes

Fullerenes are allotropes of carbon discovered in 1985 [126] that have been widely evaluated for biomedical applications, such as drug and gene delivery [127], bioimaging [128] and quenching of reactive oxygen species [128]. Analysis in zebrafish has shown that fullerenes exhibit toxicity that is related to their surface chemistry. Exposure of embryos to C_60_ or C_70_ fullerenes at 200 ppb delayed development and resulted in specific caudal fin malformation and significant pericardial edema, with >200 ppm C_60_ or C_70_ leading to 100% mortality. In contrast, exposure to >2500 ppb C_60_(OH)_24_ was required to induce fin malformations and pericardial edema, with significant mortality only observed at concentrations of >4000 ppb [129]. Another study of water soluble fullerenes indicated that positively-charged fullerenes showed enhanced toxicity compared to negatively-charged fullerenes with similar structures. Toxicity was shown to vary considerably between the negatively-charged fullerenes from very low to moderate, depending on structural features [130]. Kuznetsova et al. showed phosphatidylcholine-based phospholipid NPs containing C_60_ elicited low toxicity on zebrafish embryos [131]. Photons appear to exacerbate toxicity, since reducing light levels during exposure to C_60_ (at 200–300 ppb) significantly decreased mortality as well as the incidence of fin malformations and pericardial edema [132]. Other research has suggested that water-soluble fullerenes may protect against apoptotic cell death [130]. This was supported by a study investigating a C_60_ fullerene based derivative (dendrofullerene) containing 18 carboxylic groups, which was able to reduce radiation-induced nerve cell damage through its actions as a free-radical scavenger [133]. Multi-shell fullerene structures, known as nano-onions, demonstrate good biocompatibility, with little toxicity and homogenous biodistribution in zebrafish larvae [134].

#### 4.3.2. Carbon Nanotubes

Carbon nanotubes (CNTs) have attracted intense interest for various biomedical applications due to their distinctive chemical and physical characteristics [124]. These characteristics make CNTs promising candidates for the delivery of chemotherapeutic agents, including paclitaxel and doxorubicin, small interfering RNAs, genes and antibodies [135,136].

Pristine CNTs have been shown to have high biocompatibility, with single-walled (SW), multi-walled (MW) CNTs or carboxylated MW pristine CNTs having little effect on embryo viability and development, even at high concentrations (200 ug/mL) [137]. In another study SW CNTs functionalized by polyethylene glycol increased mortality, delayed hatching and decreased total larval length only at the highest concentration tested (1 ppm), but with no genotoxicity observed and no evidence of the nanotubes being taken up by tissues [138]. Another group found that the length of CNTs was an important determinant of toxicity with longer CNTs causing distinctive cellular and molecular changes [139].

Exposure of adult zebrafish to MW CNT (diameter 500 nm) caused reversible inflammation in the gills, but again no genotoxicity was seen [140]. Another study showed that MW CNTs were able to accumulate in zebrafish [141], while Li et al. demonstrated that exposure to CNTs can result in alterations in the brain and gonads [142].

#### 4.3.3. Carbon/Graphene Quantum Dots

**Figure 2:**
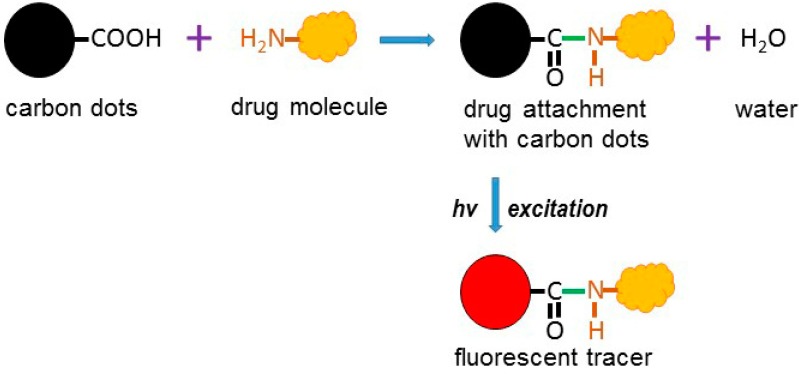
Carbon nanodot-based drug delivery and tracking. Schematic representation showing conjugation of carbon nanodots with drug molecules, and subsequent excitation to generate agents that act simultaneously as fluorescence tracers and drug delivery vehicles.

C-dots are a type of quasi-spherical carbon material with a diameter of <10 nm [143], whereas graphene QDs (GQDs) represent multiple layers of graphene with a size of <30 nm [144,145]. Both C-dots and GQDs have emerged as superior universal fluorophores due to their unique combination of excellent photostability, small size and highly tunable photoluminescence, being effective in photon-harvesting in the short-wavelength region because of π−π* transition of C=C bonds [146], with a variety of imaging applications [147]. C-dots and GQDs are also attractive for drug/gene delivery [148,149,150,151] due to their low toxicity, a consequence of the predominance of inert carbon rather than more reactive hydrogen, nitrogen and oxygen in their make-up [150,152]. However, C-dots and GQDs do contain −OH, −NH_2_, >C=O and –COOH functional groups on their surface, which increase water solubility [143], as well as assisting in covalent bond formation with antibody, chemotherapeutic agent or other biochemical entity [153,154], which can be combined with their florescence properties (Figure 2) for multi-purpose theranostic applications.

C-dots have been shown to exhibit higher biocompatibility than other NPs [55]. N-doped C-dots synthesized from BSA were well tolerated by zebrafish embryos immersed in them at concentrations of 6 mg/L and retained fluorescence for up to 2 days, highlighting their low toxicity and stable fluorescence emission [155]. In another study, embryos exposed to C-dots showed normal development at concentrations of 2 mg/L (soaking) or 1.5 mg/mL (injection). However, at higher concentrations, delayed development, inhibition of pigmentation, pericardial edema, and delayed hatching were observed. GQDs also exhibited high biocompatibility, being readily excreted from adult zebrafish without affecting growth significantly at a concentration lower than 2 mg/mL [54]. The GQDs were found to accumulate in the digestive system, while the blood, muscle and other tissue showed no obvious photoluminescence signal.

## 5. Conclusions

The nanotechnology industry is already large; it was estimated to be worth $39.2 billion in 2016 [156], and is growing rapidly. To facilitate this, efficient and effective testing of new products is essential. Zebrafish represent an excellent in vivo model for testing nanoparticle toxicity and biocompatibility. They are low cost and easy to maintain, and able to test agents efficiently via multiple routes of exposure, including directly in the water, which is especially relevant for environmental toxicology applications. In addition, specific physiological impacts can be assessed at multiple stages of development. In this review, the toxicity of different metal and carbon-based NPs has been described, identifying a range of parameters regarding exposure (concentration, route, length, life-stage, presence of other molecules) and of physiochemical properties of the NPs (size, shape, surface charge/chemistry) that impact on their biocompatibility.

There is now a growing consensus advocating the greater use of zebrafish models to reduce reliance on rodent testing for financial, ethical and biological reasons [31]. Indeed, toxicity and safety testing in zebrafish has been accepted by the Federal Drug Administration for new drug approval [25]. This will be greatly augmented by standardization of delivery protocols, methods of analysis and strains of zebrafish used. Zebrafish also have great potential for pre-clinical studies and so represent an ideal model to assist the further development of NPs as therapeutic agents, such as testing mitigation strategies when a promising NP agent exhibits toxicity.

## Figures and Tables

**Table 1:** LC_50_ toxicity testing in zebrafish embryos and adults of nanoparticles detailed in this review and selected others.

Nano Particle	Stage	LC_50_ (mg/L)	Time	Teratogenicity	Reference
Cu	eggs	24.0	48 h	Malformations, delayed hatching	[72]
	adults	4.2	48 h	N/A	[72]
		1.5	48 h	N/A	[73]
Chitosan	eggs	280	96 h	Malformations	[30]
Au	eggs	>200	48 h	None	[72]
	adults	>200	48 h	N/A	[72]
Ag	eggs	2.7	48 h	Malformations	[72]
		1.2	96 h	Malformations	[74]
	adults	2.9	48 h	N/A	[72]
Cd/Te QDs	eggs	186 (nM)	120 h	Malformations, delayed hatching	[75]
TiO_2_	eggs	>1600	48 h	Premature hatching	[72]
	adults	>1600	48 h	N/A	[72]
ZnO	eggs	3.5−9.1	120 h	None	[76]
		1.8	96 h	Delayed hatching	[77]
MgO	eggs	>3200	48 h	None	[72]
		428	96 h	Delayed hatching	[78]
	adults	140	48 h	N/A	[72]
Fe_2_O_3_	eggs	>1600	48 h	None	[72]
	adults	>1600	48 h	N/A	[72]
NiO	eggs	1700	48 h	None	[72]
	adults	760	48 h	N/A	[72]
		45	30 d	N/A	[79]
CuO	eggs	960	48 h	None	[72]
		175	48 h	None	[80]
	adults	400	48 h	N/A	[72]
Fullerene	eggs	>200	48 h	None	[72]
		1.5	96 h	Reduced hatching	[81]
	adults	>200	48 h	N/A	[72]
CNTs	eggs	>200	48 h	None	[72]
		>360	96 h	None	[82]
	adults	>200	48 h	N/A	[72]
